# Synergistic Roles of InlA, InlB and LLO in the Infection of Trigeminal Ganglion Neurons by Ovine-Derived *Listeria monocytogenes* LM90SB2

**DOI:** 10.3390/ani16091383

**Published:** 2026-04-30

**Authors:** Yue Lv, Qiuyan Deng, Ye Li, Yuxuan Lu, Jiahui Xie, Jingjing Ren, Jianjun Jiang

**Affiliations:** College of Animal Science and Technology, Shihezi University, Shihezi 832003, China; 18846914649@163.com (Y.L.); 15237377963@163.com (Q.D.); islily1215@163.com (Y.L.); 15009938623@163.com (Y.L.); xuanxiu668@163.com (J.X.)

**Keywords:** *Listeria monocytogenes*, ovine-derived strain, InlA, InlB, LLO, trigeminal ganglion, zoonotic pathogen

## Abstract

*Listeria monocytogenes* (Lm) is an important zoonotic foodborne pathogen that causes severe rhombencephalitis in ruminants. The trigeminal ganglion is a critical node for Lm invasion of the central nervous system via neural pathways. However, the roles of key virulence factors InlA, InlB, and LLO from ovine-derived Lm in trigeminal ganglion neuron infection remain unclear. In this study, LM90SB2, an ovine-derived Lm strain isolated from a sheep with encephalitis in Xinjiang, China, was used as the wild type, and its Δ*InlAB* double-gene deletion and Δ*InlABO* triple-gene deletion mutants were constructed. Primary mouse trigeminal ganglion cells (TGCs) were infected with these strains, and cell-association and invasion assays, bacterial colonization analysis, cell scratch tests, Western blotting, and qRT-PCR were performed to explore the effects of InlA, InlB, and LLO on Lm infection of TGCs and their regulatory roles in host adhesion molecules N-cadherin and NCAM1. The results showed that the wild-type LM90SB2 had significantly stronger cell-association, invasion, and colonization abilities in TGCs than the Δ*InlAB* and Δ*InlABO* mutants (*p* < 0.01 or *p* < 0.0001). LM90SB2 infection significantly upregulated the mRNA and protein expression levels of N-cadherin and NCAM1 in TGCs and enhanced TGC migration, while these effects were gradually attenuated with the sequential deletion of *InlA*, *InlB* and *LLO*. This study clarifies the synergistic roles of InlA, InlB, and LLO in mediating the infection of trigeminal ganglion neurons by ovine-derived Lm and reveals the molecular mechanism by which Lm promotes neural invasion by regulating the expression of host cell adhesion molecules. Our findings provide important experimental data for elucidating the neural invasion pathway of Lm in ruminants and lay a theoretical foundation for the development of targeted prevention and control strategies for ruminant listeriosis in veterinary clinical practices.

## Simple Summary

*Listeria monocytogenes* is a foodborne bacterium that can cause severe brain infection (rhombencephalitis) in ruminants such as sheep. This study investigated how three key bacterial factors—InlA, InlB, and LLO—help the bacterium infect trigeminal ganglion neurons, which are critical nerve cells that connect the face and mouth to the brain. Using an ovine-derived *L. monocytogenes* strain (LM90SB2) and genetically modified mutants missing one or more of these factors, we found that all three factors work together to enhance bacterial attachment, invasion, and colonization of neurons. They also increased the expression of host cell adhesion molecules (N-cadherin and NCAM1) and promoted neuronal migration, which may help the bacterium spread toward the central nervous system. These findings help explain how *L. monocytogenes* invades the nervous system in ruminants and may inform future strategies to prevent and treat listeriosis in livestock.

## 1. Introduction

*Listeria monocytogenes* (Lm) is a prominent Gram-positive, facultative intracellular bacterium commonly present in the natural environment and across the food chain, therefore being a serious food-borne pathogen. Food safety organizations’ data on surveillance can indicate that food products are contaminated with Lm to a large extent, posing a big threat to food safety [1]. In human beings and animals alike, this bacterium is able to cause infection in various systems, sharing similar clinical symptoms that include gastroenteritis, abortion, sepsis, and central nervous system (CNS) infections [2,3]. CNS infections such as brainstem encephalitis, meningoencephalitis, and meningitis are the major causes of deaths [4]. Traditionally, it has been believed that the Lm penetrates the CNS, mainly hematogenously. Nevertheless, more recent findings suggest that the mechanism by which Lm induces encephalitis in ruminants can be different than in other species so that the bacterium could induce encephalitis through retrograde transmission via trigeminal (TG) and vagus nerves as well [5]. Histological studies of sheep that have contracted the infection naturally exhibit different manifestations characterized by lesions of either side, or in the rare case, both sides of the brainstem and nerve activity in the brain [6]. The disease manifests itself on the brainstem in particular, and moves along axonal fibers or separate cranial nerve nuclei, which cannot be entirely explained by simple hematogenous infection. Lm can access peripheral nerves via the oral, pharyngeal or intestinal lining tissues after oral infection and then travel via neural pathways to the brainstem. The central nervous system is an important site of invasion for Lm with the trigeminal ganglion being the largest of the sensory ganglions in the head that is the junction between the peripheral sites and CNS [7,8]. However, whether Lm directly enters the trigeminal ganglion and subsequently damages the brainstem is yet to be decisively determined and the key virulence factors in this process are yet to be well determined.

Several virulence factors are necessary in Lm to bypass the host barriers. It is possible to summarize the process as follows: (1) adhesion and invasion by internalins A and B (InlA and InlB); (2) escape after phagosome into cytoplasm due to the synergistic effect of listeriolysin O (LLO) and Phospholipase A and B (PLcA and PLcB); and (3) cell-to-cell spread by action of internalin C (InlC) and actin assembly-inducing protein actin assembly-inducing protein (ActA) [9,10,11,12] It has been confirmed that InlA and InlB rely on each other in terms of Lm invasion of HIBCPP cell using in vitro blood–cerebrospinal fluid models of barrier [13]. InlA and InlB collaborate in the invasion of bacteria across the placental barrier in placental trophoblast epithelial cells [14]. LLO which is a pore-forming toxin mainly facilitates Lm escape by phagosomes. This has recently been suggested to be supported by cytoplasmic bacteria which facilitate cell-to-cell transmission utilizing LLO-mediated membrane perforation [15]. According to Zhang et al., LLO protein has toxic effects in the human brain microvascular endothelial cells, thus affecting the blood–brain barrier [16].

Cell adhesion molecules (like N-cadherin and NCAM1) are also involved in neuronal synapse establishment and axon guidance and their alterations in expression can modify host cell adhesion and migration, which precondition neural migration of bacteria [17,18]. However, the effects of Lm on N-cadherin and NCAM1 remain unclear.

Therefore, this study used primary mouse trigeminal ganglion cells (TGCs) as a model to investigate the roles of InlA, InlB, and LLO in Lm adhesion, invasion, and their effects on N-cadherin and NCAM1 expression. Wild-type LM90SB2, ΔInlA/InlB double-gene deletion strain (Lm90SB2ΔInlA/InlB), and ΔInlA/InlB/LLO triple-gene deletion strain (Lm90SB2ΔInlA/InlB/LLO) were used to infect TGCs. This study provides a basis for elucidating Lm CNS infection via the trigeminal nerve and offers references for research on other bacterial meningitis pathogens.

Pioneering studies from research groups in France [15,19] and Scotland [20] have laid a profound foundation for our understanding of Listeria rhombencephalitis, demonstrating the critical roles of InlA, InlB, and LLO in crossing host barriers. Building upon these well-established frameworks, we further recognize that Lm is a typical zoonotic pathogen with highly conserved virulence mechanisms across diverse mammalian hosts. While ruminants serve as the primary natural hosts for neurolisteriosis, investigating molecular pathogenesis directly in large ruminants is severely constrained by ethical considerations, economic costs, and a lack of standardized immunological tools. Consequently, the murine model has been universally adopted as the standard, economically viable, and ethical primary step for screening and verifying the pathogenic mechanisms of mutant strains before eventual validation in natural hosts [21].

## 2. Materials and Methods

### 2.1. Materials

#### 2.1.1. Animals

Specific pathogen-free (SPF) C57BL/6 mice (aged 8 weeks; half male, half female; body weight 18–22 g) were used in this study. The animals were housed in a controlled environment with a temperature of 22 ± 2 °C, relative humidity of 55 ± 10%, and a 12 h light/dark cycle. Food and water were provided ad libitum. This study was approved by the Animal Ethics Committee of Shihezi University (Approval No. A2022-18). All animal experimental procedures were strictly performed in accordance with the National Guidelines for the Care and Use of Laboratory Animals in China.

#### 2.1.2. Bacterial Strains and Cells

The wild-type *Listeria monocytogenes* strain LM90SB2 was originally isolated from a sheep with encephalitis in Xinjiang, China, and is preserved in our laboratory. The LM90Δ*InlA/InlB* (Δ*InlAB*) double-gene deletion mutant was previously constructed and characterized by our laboratory [22]. Based on the Δ*InlAB* background, the LM90Δ*InlA/InlB/LLO* (Δ*InlABO*) triple-gene deletion mutant was newly generated in this study using a homologous recombination strategy. Briefly, the upstream and downstream homologous arms of the llo gene were amplified and cloned into a temperature-sensitive shuttle vector. The recombinant plasmid was electroporated into the Δ*InlAB* strain, followed by chromosomal integration at 41 °C and subsequent excision at 30 °C to create the in-frame deletion. The specific primers used for Δ*InlABO* construction are listed in Supplementary Table S1.

To facilitate visualization, the wild-type and mutant strains were labeled with superfolder green fluorescent protein (sfGFP). This was achieved by electroporating the integrative plasmid pIMKC-sfgfp(constructed in our laboratory; Addgene, Watertown, MA, USA) into the respective strains. The sfgfp gene was stably integrated into the bacterial chromosome at the PSA tRNA^Arg-*attBB*^ site, and positive transformants were selected on BHI agar (Hopebio, Qingdao, China) with 10 µg/mL chloramphenicol (Solarbio, Beijing, China). To ensure that genetic manipulations did not impair bacterial fitness, the growth kinetics of all strains in BHI medium were verified. Furthermore, to ensure that these genetic manipulations did not impair bacterial fitness, the comprehensive growth kinetics of all strains in BHI medium were systematically evaluated over a 14-hour period. As demonstrated by the full-cycle growth curves (Supplementary Figure S1), which are consistent with previously established reports [22,23], neither the sequential deletion of *inlA*, *inlB*, and *llo*, nor the introduction of the integrative sfGFP plasmid, significantly altered the basal growth rates or stationary phase capacities compared to the parental wild-type strain.

Primary trigeminal ganglion cells (TGCs) were isolated in this study and cultured in Neurobasal medium (Gibco, Waltham, MA, USA) supplemented with 10% fetal bovine serum (FBS, ExCell Bio, Shanghai, China) and 2% B-27 supplement (Gibco) at 37 °C with 5% CO_2_.

### 2.2. Methods

The isolated primary TGCs were randomly allocated to each experimental and control group via a random number table method. A single-blind strategy was implemented during data collection and statistical analysis, where the researchers conducting the detection were unaware of the group allocation to eliminate subjective bias.

#### 2.2.1. Isolation and Identification of Primary TGCs

Isolation and Culture of Primary TGCs: Six to seven mice were euthanized, and their heads were disinfected with 75% ethanol. The skin was removed, and the cranium was opened via a midline incision. After carefully removing the brain, the cranial base was exposed. The bilateral trigeminal ganglia, which are characterized by their distinct semilunar shape, were identified within the Meckel’s cave on the petrous part of the temporal bone. The overlying dura mater was meticulously removed using micro-dissection forceps. The identified ganglia were then aseptically dissected and placed in pre-cooled PBS (Solarbio, Beijing, China) containing 1% penicillin–streptomycin (Gibco, Waltham, MA, USA). Ganglia were minced and centrifuged (1500 r/min, 5 min). After discarding the supernatant, Type IA collagenase (1 mg/mL, Gibco) and 0.25% trypsin (Gibco) were added sequentially for digestion at 37 °C. Complete medium was added to stop digestion, and cells were dispersed by pipetting. The cell suspension was filtered through a 70 μm cell strainer and centrifuged (1500 r/min, 10 min). Cells were seeded onto D-polylysine (Solarbio, Beijing, China)-coated plates. Half of the medium was replaced on day 3, and cells were cultured for 5–7 days before experiments.

Immunofluorescence Identification of Primary TGCs: TGCs were seeded on coverslips in 24-well plates (2 × 10^4^ cells/well). After adherence, cells were fixed with 4% paraformaldehyde (Solarbio) at 4 °C for 30 min. Cells were treated with blocking buffer containing 0.5% Triton X-100 (Solarbio) and 10% serum for 2 h. Primary antibody (β-Tubulin-III, 1:100, Proteintech, Rosemont, IL, USA) was added and incubated at 4 °C overnight. After washing, secondary antibody (Goat anti-Rabbit IgG (H+L) Cross-Adsorbed Secondary Antibody Alexa Fluor 488, 1:500, Proteintech) was incubated in the dark at room temperature for 2 h. Nuclei were stained with DAPI (Solarbio), and coverslips were mounted with Fluoromount-G (SouthernBiotech, Birmingham, AL, USA). Images were captured using a fluorescence microscope.

Throughout all infection experiments, including the 24 h prolonged infections for molecular analyses, the cellular morphological integrity of the TGCs was closely monitored via phase-contrast microscopy to ensure the cells remained intact and firmly adherent prior to sample collection. This morphological stability aligns with established neurobiological consensus that fully differentiated, post-mitotic neurons possess highly restricted apoptotic pathways and distinct resilience against severe intracellular stress or pathogen burdens compared to actively dividing somatic cells [24,25].

#### 2.2.2. Cell-Association and Invasion Assays

Infection Conditions: TGCs were cultured in 6-well plates (approximately 1 × 10^6^ cells/well) until 80–90% confluent and infected at a multiplicity of infection (MOI) of 100 with LM90, Δ*InlAB*, or Δ*InlABO* strains in antibiotic-free medium. Plates were incubated at 37 °C for 1 h with gentle shaking. Throughout all infection experiments, including the 24 h prolonged infections for molecular analyses, the cellular viability and morphological integrity of the TGCs were closely monitored via phase-contrast microscopy to ensure the cells remained healthy, intact, and firmly adherent prior to sample collection. Each group had ≥ 3 technical replicates, and experiments were independently repeated ≥ 3 times.

Cell-association Assay: Following a 1-hour infection period, the cells were rinsed twice with PBS. Subsequently, 1 mL of 0.2% Triton X-100 was introduced to each well at room temperature for a duration of 5 min to induce cell lysis. The resultant lysate was gathered, subjected to a 10-fold serial dilution, and then spread onto BHI agar plates (2–3 plates for each dilution). The plates were incubated overnight at 37 °C, after which CFUs were quantified to assess “cell-associated bacteria” (comprising both externally cell-associated and internalized bacteria).

Invasion Assay (Gentamicin Protection Assay): After an hour of infection, the cells were washed twice using PBS, and a medium containing 0.1 g/L gentamicin was added for 1 h at 37 °C to eliminate any extracellular bacteria. The cells were then washed twice with PBS to eliminate the antibiotics. Following this, 1 mL of 0.2% Triton X-100 was used to lyse the cells, and the lysate was serially diluted and plated on BHI plates. After an overnight incubation at 37 °C, CFUs were counted to evaluate “invaded bacteria”.

#### 2.2.3. Immunofluorescence Bacterial Imaging

TGCs were seeded on Matrigel-treated coverslips in 24-well plates at a density of approximately 2 × 10^4^ cells/well. Once adhesion occurred, the cells underwent infection using GFP-labeled strains at a MOI of 1 for a duration of 24 h. The cells were fixed using 4% PFA for 15 min and subsequently washed with PBS. To permeabilize the cells, a solution of 0.1% Triton X-100 was used for 10 min, followed by PBS washing, and blocking with 5% BSA for 1 h at ambient temperature. The cells were then treated with an antibody (CoraLite^®^594-conjugated Beta Actin Monoclonal Antibody, diluted 1:100, Proteintech) in a dark environment for 2 h, after which they were washed with PBS, and the nuclei were labeled using DAPI for 5 min. Finally, the coverslips were mounted and examined using a confocal microscope. We quantified the cell-associated bacteria as visualized in the immunofluorescence images. Although the use of confocal laser scanning microscopy allowed for clear visualization of bacteria localized in close proximity to the host cell nuclei and within the same focal plane as the cytoplasm—strongly suggesting an intracellular location—we have adopted the term ‘cell-associated’ to remain terminologically rigorous, encompassing all bacteria firmly associated with the TGCs at this 24 h time point. The number of internalized GFP-positive bacteria within individual TGCs was counted using ImageJ software (version 1.53t, NIH, Bethesda, MD, USA). For each experimental group, at least five randomly selected microscopic fields of view were analyzed to calculate the average total bacterial count per field.

#### 2.2.4. Cell Scratch Assay

TGCs were seeded in 6-well plates at 1 × 10^6^ cells/well. When cells reached appropriate density, they were infected at MOI = 100. The scratch assay was performed as described by Wang et al. (2024) [26]. Scratch areas were observed at 0 and 24 h under an inverted microscope to record cell migration. Uninfected cells cultured in the same medium served as the negative control (NC). Each group had 3 replicates. The migration area was quantified using ImageJ software. The cell migration rate was calculated according to the following formula: Migration Rate (%) = (A_0_ − At)/A_0_ × 100%, where A_0_ represents the initial scratch area at 0 h, and At represents the remaining scratch area at 24 h post-infection.

#### 2.2.5. Western Blot Analysis of N-Cadherin and NCAM1

TGCs were cultured to reach 80–90% confluence and subsequently infected with strains at a MOI of 100 for 24 h. Total protein from the treated TGCs was extracted using RIPA lysis buffer supplemented with 1% protease and phosphatase inhibitor cocktail (Solarbio, Beijing, China). The cell lysates were incubated on ice for 30 min and then centrifuged at 12,000× *g* for 15 min at 4 °C to collect the supernatant. Protein concentrations were determined using a BCA protein assay kit. Proteins were extracted, and their concentrations were quantified utilizing the BCA assay. Equivalent quantities of protein were resolved using 8% SDS-PAGE and then transferred onto PVDF membranes. The membranes were blocked for 1 h at room temperature with 5% skim milk and incubated overnight at 4 °C with primary antibodies (Anti-N-Cadherin Antibody, 1:1000, Abcam; Anti-NCAM1 Antibody [EP2567Y], 1:1000, Abcam, Cambridge, UK). Following a washing step, the membranes were treated with a secondary antibody (Goat anti-Rabbit IgG (H+L), 1:5000, Proteintech) for 1 h at room temperature. Enhanced chemiluminescence (ECL) was employed for signal detection. GAPDH acted as the loading control. Band intensities were evaluated using ImageJ software. Each experimental group consisted of at least three technical replicates, and the experiments were conducted independently a minimum of three times. The uncropped raw Western blot data of this study contained five bands, among which one band was the preliminary experimental data of another Listeria strain that was not incorporated into the experimental design and result analysis of this study. Thus, this band was not presented in the figures of the manuscript, and only the four valid bands corresponding to the experimental groups of this study were used for statistical analysis. The protein bands were visualized and subsequently quantified using ImageJ software (version 1.53t, National Institutes of Health, Bethesda, MD, USA) [27]. Specifically, the integrated density of each target protein band was measured, and the relative expression levels were normalized to the corresponding GAPDH internal control.

#### 2.2.6. qRT-PCR Analysis for N-Cadherin and NCAM1

TGCs were subjected to infection with strains at an MOI of 100 for a duration of 24 h. Total RNA was isolated employing an RNA extraction kit (TransGen Biotech, Beijing, China), and quantitative real-time polymerase chain reaction (qRT-PCR) was carried out following the manufacturer’s guidelines (TransGen Biotech). The primer sequences are detailed in Table 1. GAPDH served as the internal reference gene. In rigorous compliance with the MIQE guidelines, all qRT-PCR primers were thoroughly validated prior to the formal experiments. Primer specificity was confirmed by the presence of a single, distinct peak in the melt-curve analysis. Furthermore, amplification efficiencies were evaluated using standard curves generated from serial dilutions of cDNA. All primers demonstrated highly acceptable performance parameters, with amplification efficiencies ranging from 95% to 105% and correlation coefficients (R^2^) > 0.99. Relative expression levels of the genes were computed using the 2^−ΔΔCt^ approach. This experiment was replicated three times.

#### 2.2.7. Statistical Analysis

All data were examined employing SPSS version 20.0 (SPSS Inc., Chicago, IL, USA). For comparisons across multiple groups, a one-way ANOVA was applied. To compare two groups, independent sample *t*-tests were conducted. A significance level of * *p* < 0.05 was deemed statistically relevant. The thresholds were set as * *p* < 0.05, ** *p* < 0.01, *** *p* < 0.001, and **** *p* < 0.0001. Graphs were generated using GraphPad Prism 9 (La Jolla, CA, USA).

## 3. Results

### 3.1. Identification of Primary TGCs

**Figure 1:**
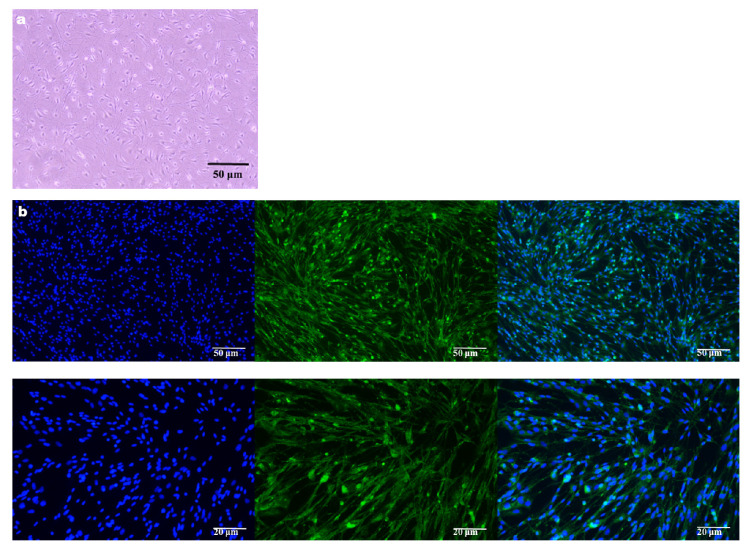
Isolation and identification of primary mouse TGCs. (**a**) In vitro culture morphology of primary mouse TGCs observed under light microscopy; (**b**) Immunofluorescence identification of the neuron-specific marker β-III Tubulin (Tuj1, green) in TGCs, with nuclei counterstained with DAPI (blue). Scale bars are indicated in the figures.

Primary TGCs were successfully isolated and cultured, showing good adherent growth under microcopy. Immunofluorescence staining confirmed positive expression of the neuron-specific marker β-III Tubulin (Tuj1) in the cytoplasm of TGCs (Figure 1).

### 3.2. Cell-Association and Invasion Assay Results

**Figure 2:**
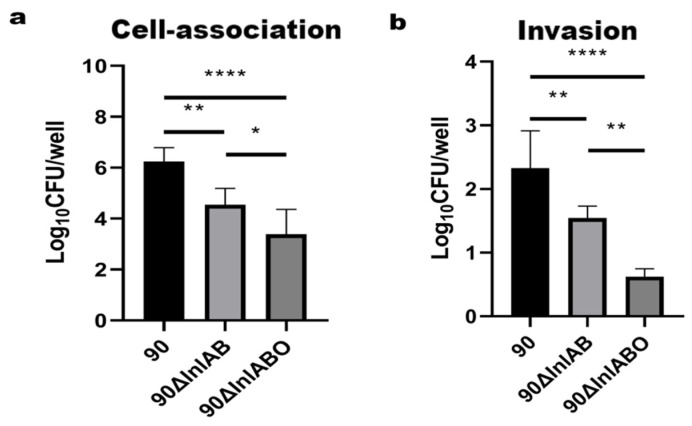
Role of InlA and InlB in the infection of primary TGCs. (**a**) Cell-associated Lm (including both surface-cell-associated and internalized bacteria) quantified 1 h post-infection. (**b**) Invaded Lm (internalized bacteria only) quantified after gentamicin treatment. Cell-association assay results are expressed as Log_10_ CFU/well. * *p* < 0.05, ** *p* < 0.01, **** *p* < 0.0001.

Cell-association assay results showed that the LM90 group was significantly higher than the Δ*InlAB* group (*p* < 0.01) and the Δ*InlABO* group (*p* < 0.0001). The Δ*InlAB* group was higher than the Δ*InlABO* group (*p* < 0.05) (Figure 2a). Invasion assay results showed that the LM90 group was significantly higher than the Δ*InlAB* group (*p* < 0.01) and the Δ*InlABO* group (*p* < 0.0001). The Δ*InlAB* group was significantly higher than the Δ*InlABO* group (*p* < 0.01) (Figure 2b).

### 3.3. Immunofluorescence Bacterial Imaging Results

**Figure 3:**
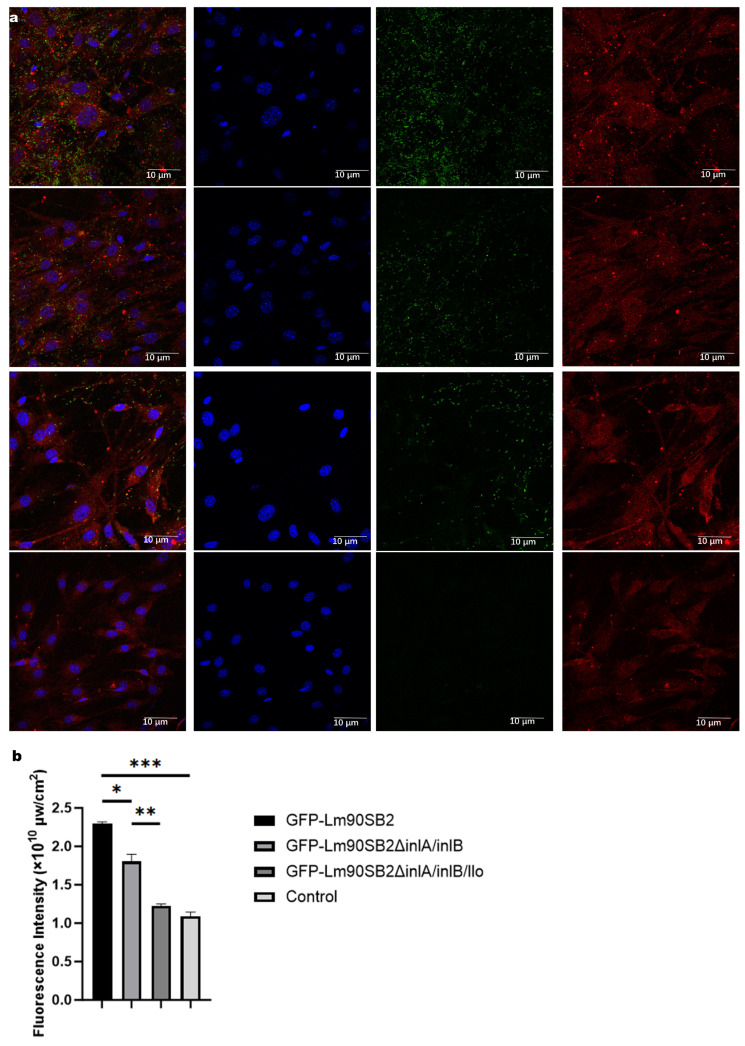
Colonization of GFP-labeled Lm strains in primary mouse TGCs. (**a**) Representative confocal microscopy images showing the colonization of wild-type LM90, Δ*InlAB*, and Δ*InlABO* strains (green) in TGCs (β-Actin, red; DAPI, blue); (**b**) Quantitative analysis of total bacterial counts within TGCs. NC represents uninfected cells cultured in the same medium. Data are expressed as mean ± SD. * *p* < 0.05, ** *p* < 0.01, *** *p* < 0.001.

Immunofluorescence imaging combined with bacterial counting showed colonization of GFP-labeled LM90, Δ*InlAB*, and Δ*InlABO* strains in TGCs. Confocal microscopy revealed extensive GFP fluorescence (green) co-localized with cells (β-Actin, red; DAPI, blue) in the LM90 group, indicating dense bacterial colonization. GFP signals decreased in the Δ*InlAB* group and further reduced in the Δ*InlABO* group. No GFP signal was observed in the negative control. Statistical analysis of bacterial counts showed that the LM90 group had significantly higher total bacterial counts than the Δ*InlAB* group (*p* < 0.001) and the Δ*InlABO* group (*p* < 0.0001). The Δ*InlAB* group had significantly higher counts than the Δ*InlABO* group (*p* < 0.001) (Figure 3).

### 3.4. Cell Scratch Assay Results

**Figure 4:**
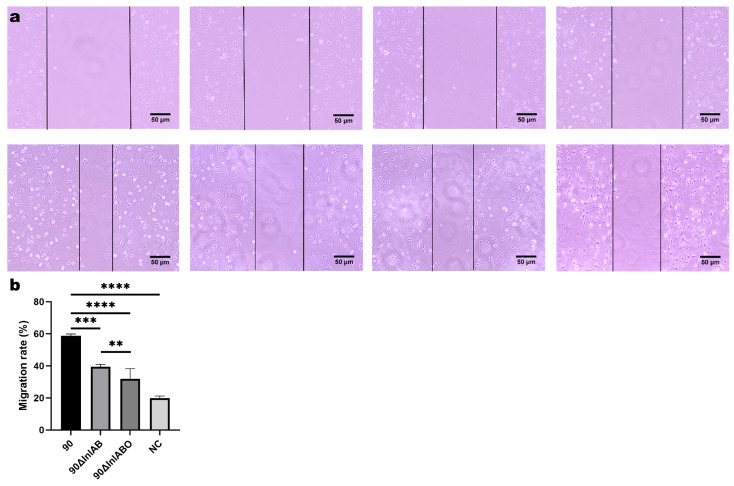
Effect of Lm infection on the migration capacity of primary mouse TGCs. (**a**) Representative images of the cell scratch assay at 0 h and 24 h post-infection; (**b**) Statistical analysis of the migration rates (%) for each experimental group. NC represents uninfected cells cultured in the same medium. ** *p* < 0.01, *** *p* < 0.001, **** *p* < 0.0001.

The scratch assay showed that the LM90 group had significantly higher migration rates than the Δ*InlAB* group (*p* < 0.001), Δ*InlABO* group (*p* < 0.0001), and NC group (*p* < 0.0001). The Δ*InlAB* group had significantly higher migration rates than the Δ*InlABO* group (*p* < 0.01) and NC group (*p* < 0.001). The Δ*InlABO* group had higher migration rates than the NC group (*p* < 0.01) (Figure 4).

### 3.5. Transcriptional and Translational Expression of N-Cadherin and NCAM1

**Figure 5:**
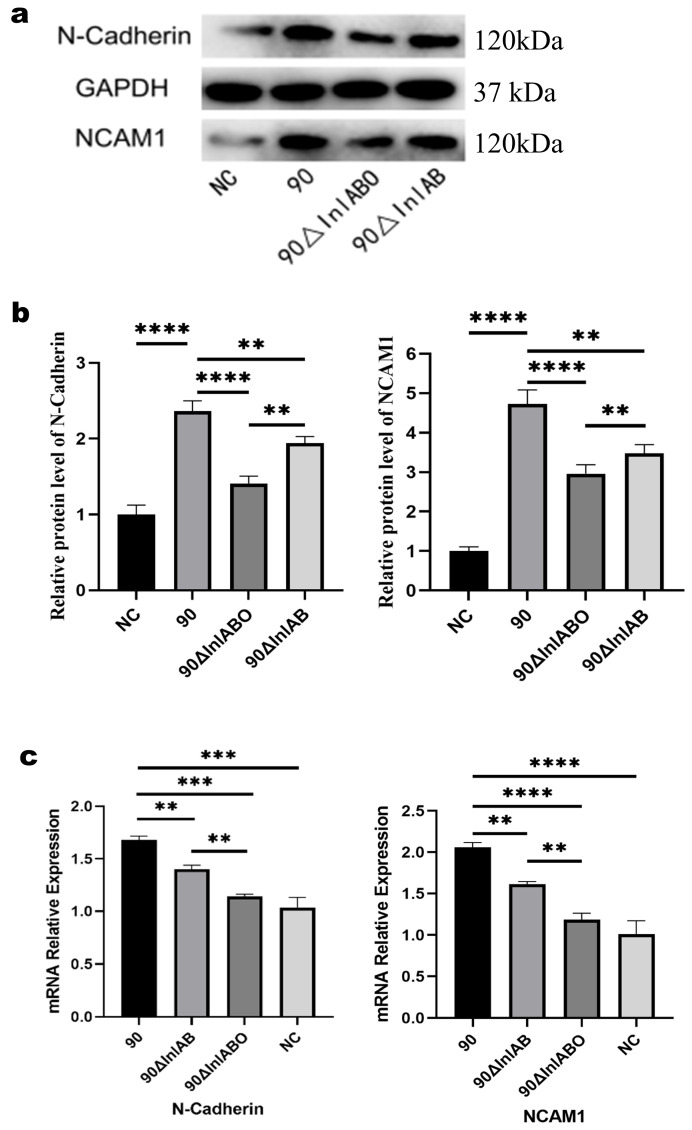
Transcriptional and translational expression profiles of N-Cadherin and NCAM1 in primary mouse TGCs infected with different Lm strains. (**a**) Representative WB bands of N-Cadherin and NCAM1 protein expression; GAPDH was used as the internal loading control. (**b**) Quantitative analysis of relative protein expression levels (normalized to GAPDH). (**c**) Relative mRNA expression levels of N-cadherin and NCAM1 determined by qRT-PCR (normalized to GAPDH). NC represents uninfected cells cultured in the same medium. Data are presented as mean ± SD from three independent experiments. ** *p* < 0.01, *** *p* < 0.001, **** *p* < 0.0001.

To systematically evaluate the impact of Lm infection on host adhesion molecules, both qRT-PCR and WB analyses were conducted to determine the mRNA and protein expression levels of N-Cadherin and NCAM1 in primary mouse TGCs. The integrated results demonstrated that wild-type LM90SB2 infection robustly upregulated both the mRNA transcripts and the corresponding protein levels of N-Cadherin and NCAM1 compared to the NC group (*p* < 0.01 to *p* < 0.0001). Conversely, this infection-induced upregulation was significantly attenuated in the Δ*InlAB* group and further diminished in the Δ*InlABO* group across both transcriptional and translational levels. Notably, no significant difference in mRNA expression was observed between the Δ*InlABO* mutant and the NC group (ns). These consistent molecular profiles (Figure 5) indicate that the sequential deletion of *InlAB* and *llo* progressively impairs the pathogen’s capacity to induce the expression of these critical adhesion molecules.

## 4. Discussion

Listeria is an important pathogen with both foodborne and neuroinvasive properties. Its invasion of the CNS is a key step in causing disease in animals and humans, and the trigeminal pathway has been confirmed as an important route for retrograde infection of the brainstem [28]. While previous landmark studies have broadly elucidated the in vivo dissemination of Lm, our study corroborates these findings but innovates methodologically by utilizing highly pure primary murine trigeminal ganglion cells (TGCs) in vitro. This sophisticated cellular approach provides a more direct and unconfounded molecular perspective on how the pathogen manipulates neural adhesion molecules (N-Cadherin and NCAM1) during the subsequent steps of retrograde axonal transport after initial nerve invasion, bridging a specific gap left by earlier systemic models.

Our results show that wild-type LM90 has significantly stronger adhesion, invasion, and colonization abilities in TGCs than Δ*InlAB* and Δ*InlABO* mutants (*p* < 0.01 or *p* < 0.0001), with a gradient decrease as more virulence factors are deleted. This finding is consistent with previous studies that *InlAB* proteins are core virulence factors of Lm, mediating invasion by binding to host cell surface receptors [29,30]. In neuronal cells, deletion of these genes directly weakens bacterial host recognition and invasion efficiency. Notably, TGCs, as an important component of the peripheral nervous system, show functional abnormalities after pathogen invasion that are closely related to clinical neurological symptoms in animals. Ruminant listeriosis typically manifests as brainstem encephalitis, often accompanied by sensory abnormalities and motor disorders in trigeminal nerve-innervated areas [31]. The efficient colonization of TGCs by LM90 provides in vitro evidence for explaining the neural dissemination pathway in clinical cases. The significant decrease in colonization ability of mutant strains indicates that *InlAB* and LLO genes not only regulate Lm interaction with epithelial cells but are also key molecular bases for breaking through neural cell barriers. This finding expands the functional understanding of *InlAB* and LLO virulence factors in neural invasion. It is widely recognized that the interaction between InlA and murine E-Cadherin is highly inefficient due to a species-specific amino acid mismatch [32]. However, primary TGCs, being sensory neurons, intrinsically lack epithelial E-Cadherin and predominantly express neural adhesion molecules. The significant reduction in cellular invasion following InlA deletion in our mouse TGC model suggests an intriguing paradigm, as the receptor for InlB (c-Met) is highly conserved in mice [15]. In the present study, we utilized a Δ*InlAB* double mutant to assess these virulence factors. However, because single-deletion mutants of inlA or inlB were not evaluated, we cannot definitively attribute a specific function to InlA during the cell-association and invasion of TGCs. It is highly plausible that the observed reduction in cellular invasion is predominantly or solely driven by the deletion of inlB in the double mutant, and the consequent loss of interaction with the c-Met receptor. To precisely elucidate whether InlA plays any functional role in peripheral neural infection, future investigations utilizing individual single-gene deletion mutants are strictly required. While the use of ovine primary TGCs would perfectly mimic the natural infection, the extreme technical difficulties in primary cell extraction and the lack of standardized specific antibodies make the murine model an indispensable tool for these fundamental mechanistic explorations [28]. Moreover, previous neurobiological studies have documented well that the c-Met receptor, the specific target for InlB, is abundantly and constitutively expressed in rodent peripheral sensory neurons [33,34]. This native high expression of c-Met in TGCs provides a robust physiological basis for the substantial involvement of InlB in facilitating neuronal invasion.

Cell adhesion molecules (CAMs) play a dual role in pathogen–host cell recognition, binding, and signal transduction, participating in host defense responses and being exploited by pathogens as invasion targets [35,36]. NCAM1, a member of the immunoglobulin superfamily, is involved in neuronal adhesion and differentiation and plays a role in immune regulation during pathogen infection. For example, in Mycobacterium tuberculosis infection, NCAM1 can regulate humoral immune responses. N-Cadherin, a member of the cadherin family, plays a central role in neural cell connection formation and maintenance, and its abnormal expression can directly affect neuronal migration and tissue integrity [37,38,39,40]. This study found that LM90 infection significantly upregulated N-Cadherin and NCAM1 mRNA and protein expression in TGCs (*p* < 0.0001), while *InlAB* gene deletion significantly weakened this upregulation effect in a gene dose-dependent manner. Furthermore, upregulation of both adhesion molecules coincided with high colonization ability of Lm, suggesting they may mediate Lm interaction with TG cells. On one hand, high expression of adhesion molecules may provide more cell surface binding sites for Lm, promoting bacterial colonization. It should be noted that current literature lacks direct evidence classifying N-Cadherin and NCAM1 as primary binding receptors for Lm virulence factors. Instead of providing direct binding sites, we hypothesize that their robust overexpression likely induces cytoskeletal rearrangements and enhances cellular motility, thereby indirectly facilitating the internalization and trans-neural dissemination of the pathogen [40,41]. On the other hand, abnormal expression may interfere with normal physiological functions of TG cells, consistent with subsequent cell migration assay results.

The scratch assay showed that the migration rate of TGCs in the LM90 group was significantly higher than in Δ*InlAB*, Δ*InlABO*, and NC groups (*p* < 0.0001). The migration rates of Δ*InlAB* and Δ*InlABO* groups decreased sequentially but were still higher than the NC group (*p* < 0.01 or *p* < 0.001). This suggests that LM90 infection significantly enhances TGC migration, and deletion of *InlAB*-related genes gradiently weakens this effect. At the molecular level, this phenotype is highly related to changes in N-Cadherin and NCAM1 expression. In this study, upregulation of these adhesion molecules by LM90 infection not only enhances intercellular adhesion but also regulates neuronal migration. N-Cadherin participates in maintaining cell connections during neural development and can promote directional cell migration through “adhesion traction” mechanisms. Chen et al. (2024) [41] confirmed that high NCAM1 expression activates Rho family GTPase signaling pathways, directly regulating cytoskeleton rearrangement and enhancing cell motility. LM90 upregulates these molecules through InlA and InlB, essentially hijacking host cell migration regulation mechanisms to confer stronger motility. From a veterinary clinical perspective, abnormal enhancement of TGC migration has clear pathological significance. The trigeminal ganglion is a key node between peripheral and central nerves. Enhanced migration of infected TGCs may serve as a “carrier” for Lm dissemination in neural tissues, carrying bacteria toward the CNS (e.g., brainstem). This corresponds to the classic infection route of “retrograde invasion of the brainstem via the trigeminal nerve” in ruminant listeriosis. The decreased migration ability due to InlA and InlB gene deletion functionally confirms the “boosting role” of these virulence genes in Lm neural dissemination, providing experimental basis for clinical strategies targeting InlA and InlB to block pathogen neural spread.

## 5. Conclusions

This study, using primary mouse TGCs as a model, systematically elucidated the important and synergistic roles of *Listeria monocytogenes* virulence factors InlA, InlB, and LLO in infecting trigeminal neurons. By regulating the expression of host cell-association molecules N-cadherin and NCAM1, these virulence factors activate cell migration-related signaling pathways, creating conditions for bacterial dissemination along neural pathways. These results have important practical significance for the prevention and control of listeriosis in ruminant breeding industry, especially in pastoral areas with high incidence of sheep encephalitis.

## Figures and Tables

**Table 1:** Primer sequences for qRT-PCR.

Gene Name	Forward Primer Sequence (5′ → 3′)	Reverse Primer Sequence (5′ → 3′)	Amplicon Size (bp)
GAPDH	TGTGTCCGTCGTGGATCTGA	TGGTGAAGACGCCAGTGGA	120
N-cadherin	TCCACCAACAGCAGCATAAC	TTCCTCTTCCTCCTCCTCTTTC	137
NCAM1	CCTATGGGATTACCCTTCTCAAC	CTCGCTTGTCCAGATAGTAACC	111

## Data Availability

Data are contained within the article.

## Supplementary Materials

The following supporting information can be downloaded at: https://www.mdpi.com/article/10.3390/ani16091383/s1, Figure S1: Growth kinetics of the *Listeria monocytogenes* strains used in this study; Table S1: Primer sequences used for the construction and verification of the InlABO mutant strain.

## Abbreviations

The following abbreviations are used in this manuscript:

Lm	*Listeria monocytogenes*
TGCs	Trigeminal ganglion cells
CNS	Central nervous system
InlA	Internalin A
InlB	Internalin B
LLO	Listeriolysin O
PlcA	Phospholipase A
PlcB	Phospholipase B
ActA	Actin assembly-inducing protein
InlC	Internalin C
MOI	Multiplicity of infection
CFUs	Colony-forming units
PFA	Paraformaldehyde
BSA	Bovine serum albumin
FBS	Fetal bovine serum
PBS	Phosphate-buffered saline
SDS-PAGE	Sodium dodecyl sulfate-polyacrylamide gel electrophoresis
PVDF	Polyvinylidene fluoride
ECL	Enhanced chemiluminescence
qRT-PCR	quantitative real-time polymerase chain reaction
BCA	Bicinchoninic acid
N-cadherin	Neural cadherin
NCAM1	Neural cell adhesion molecule 1
β-Tubulin-III	beta-Tubulin isotype III
Tuj1	Neuron-specific class III β-tubulin
SPF	Specific pathogen-free

## References

[1] Li X., Zheng J., Zhao W., Wu Y. (2024). Prevalence of *Listeria monocytogenes* in Milk and Dairy Product Supply Chains: A Global Systematic Review and Meta-analysis. Foodborne Pathog. Dis..

[2] Končurat A., Sukalić T. (2024). Listeriosis: Characteristics, Occurrence in Domestic Animals, Public Health Significance, Surveillance and Control. Microorganisms.

[3] Sotohy S.A., Elnaker Y.F., Omar A.M., Alm Eldin N.K., Diab M.S. (2024). Prevalence, antibiogram and molecular characterization of *Listeria monocytogenes* from ruminants and humans in New Valley and Beheira Governorates, Egypt. BMC Vet. Res..

[4] Koopmans M.M., Brouwer M.C., Vázquez-Boland J.A., van de Beek D. (2023). Human Listeriosis. Clin. Microbiol. Rev..

[5] Bagatella S., Tavares-Gomes L., Oevermann A. (2022). *Listeria monocytogenes* at the interface between ruminants and humans: A comparative pathology and pathogenesis review. Vet. Pathol..

[6] Oevermann A., Di Palma S., Doherr M.G., Abril C., Zurbriggen A., Vandevelde M. (2010). Neuropathogenesis of naturally occurring encephalitis caused by *Listeria monocytogenes* in ruminants. Brain Pathol..

[7] Senay T.E., Ferrell J.L., Garrett F.G., Albrecht T.M., Cho J., Alexander K.L., Myers-Morales T., Grothaus O.F., D’Orazio S.E.F. (2020). Neurotropic Lineage III Strains of *Listeria monocytogenes* Disseminate to the Brain without Reaching High Titer in the Blood. mSphere.

[8] Wei P., Bao R., Fan Y. (2020). Brainstem Encephalitis Caused by *Listeria monocytogenes*. Pathogens.

[9] Schnupf P., Zhou J., Varshavsky A., Portnoy D.A. (2007). Listeriolysin O secreted by *Listeria monocytogenes* into the host cell cytosol is degraded by the N-end rule pathway. Infect. Immun..

[10] Disson O., Lecuit M. (2012). Targeting of the central nervous system by *Listeria monocytogenes*. Virulence.

[11] Dowd G.C., Mortuza R., Bhalla M., Van Ngo H., Li Y., Rigano L.A., Ireton K. (2020). *Listeria monocytogenes* exploits host exocytosis to promote cell-to-cell spread. Proc. Natl. Acad. Sci. USA.

[12] Fields M., Spencer A., Mason S.J., Phelps C.C., Zhang X., Amer A., Seveau S. (2025). Role of listeriolysin O and phospholipases C in *L. monocytogenes* intercellular protrusion dynamics, resolution, and autophagy avoidance. mBio.

[13] Vázquez-Boland J.A., Kuhn M., Berche P., Chakraborty T., Domínguez-Bernal G., Goebel W., González-Zorn B., Wehland J., Kreft J. (2001). Listeria pathogenesis and molecular virulence determinants. Clin. Microbiol. Rev..

[14] Gründler T., Quednau N., Stump C., Orian-Rousseau V., Ishikawa H., Wolburg H., Schroten H., Tenenbaum T., Schwerk C. (2013). The surface proteins InlA and InlB are interdependently required for polar basolateral invasion by *Listeria monocytogenes* in a human model of the blood-cerebrospinal fluid barrier. Microbes Infect..

[15] Disson O., Grayo S., Huillet E., Nikitas G., Langa-Vives F., Dussurget O., Ragon M., Le Monnier A., Babinet C., Cossart P. (2008). Conjugated action of two species-specific invasion proteins for fetoplacental listeriosis. Nature.

[16] Zhang T., Bae D., Wang C. (2015). Listeriolysin O mediates cytotoxicity against human brain microvascular endothelial cells. FEMS Microbiol. Lett..

[17] Dash S., Duraivelan K., Samanta D. (2021). Cadherin-mediated host-pathogen interactions. Cell. Microbiol..

[18] Moreland T., Poulain F.E. (2022). To Stick or Not to Stick: The Multiple Roles of Cell Adhesion Molecules in Neural Circuit Assembly. Front. Neurosci..

[19] Jin Y., Dons L., Kristensson K., Rottenberg M.E. (2001). Neural route of cerebral *Listeria monocytogenes* murine infection: Role of immune response mechanisms in controlling bacterial neuroinvasion. Infect. Immun..

[20] Low J.C., Donachie W. (1997). A review of *Listeria monocytogenes* and listeriosis. Vet. J..

[21] Lecuit M. (2020). *Listeria monocytogenes*, a model in infection biology. Cell. Microbiol..

[22] Ren J.-J., Yang M.-W., Wang P.-Y., Jiang J.-J., Yan G.-Q. (2018). Construction and characterization of LM90-Δ*inlA* and LM90- Δ*inlB*/Δ*inlA* mutant strains of *Listeria monocytogenes*. Chin. J. Prev. Vet. Med..

[23] Deng Q.-Y., Qin H., Lv Y., Xie J.-H., Wang J., Ma X., Ren J.-J., Wang P.-Y., Jiang J.-J. (2026). Construction of complementary strains of Listeria monocytogenes inlA/inlB/llo deletion strains and their partial biological characteristics. Heilongjiang Anim. Sci. Vet. Med..

[24] Hollville E., Romero S.E., Deshmukh M. (2019). Apoptotic cell death regulation in neurons. FEBS J..

[25] Annis R.P., Swahari V., Nakamura A., Xie A.X., Hammond S.M., Deshmukh M. (2016). Mature neurons dynamically restrict apoptosis via redundant premitochondrial brakes. FEBS J..

[26] Wang Z., Du J., Ma W., Diao X., Liu Q., Liu G. (2024). Bacteriocins attenuate *Listeria monocytogenes*-induced intestinal barrier dysfunction and inflammatory response. Appl. Microbiol. Biotechnol..

[27] Schneider C.A., Rasband W.S., Eliceiri K.W. (2012). NIH Image to ImageJ: 25 years of image analysis. Nat. Methods.

[28] Chevee V., Hullahalli K., Dailey K.G., Guereca L., Zhang C., Waldor M.K., Portnoy D.A. (2024). Temporal and spatial dynamics of *Listeria monocytogenes* central nervous system infection in mice. Proc. Natl. Acad. Sci. USA.

[29] Pentecost M., Kumaran J., Ghosh P., Amieva M.R. (2010). *Listeria monocytogenes* internalin B activates junctional endocytosis to accelerate intestinal invasion. PLoS Pathog..

[30] Gyanwali G.C., Herath T.U.B., Gianfelice A., Ireton K. (2022). *Listeria monocytogenes* Co-Opts the Host Exocyst Complex To Promote Internalin A-Mediated Entry. Infect. Immun..

[31] Precht C., Vermathen P., Henke D., Staudacher A., Lauper J., Seuberlich T., Oevermann A., Schweizer-Gorgas D. (2020). Correlative Magnetic Resonance Imaging and Histopathology in Small Ruminant Listeria Rhombencephalitis. Front. Neurol..

[32] Lecuit M., Dramsi S., Gottardi C., Fedor-Chaiken M., Gumbiner B., Cossart P. (1999). A single amino acid in E-cadherin responsible for host specificity towards the human pathogen *Listeria monocytogenes*. EMBO J..

[33] Hashimoto N., Yamanaka H., Fukuoka T., Dai Y., Obata K., Mashimo T., Noguchi K. (2001). Expression of HGF and cMet in the peripheral nervous system of adult rats following sciatic nerve injury. Neuroreport.

[34] Zheng L.F., Wang R., Yu Q.P., Wang H., Yi X.N., Wang Q.B., Zhang J.W., Zhang G.X., Xu Y.Z. (2010). Expression of HGF/c-Met is dynamically regulated in the dorsal root ganglions and spinal cord of adult rats following sciatic nerve ligation. Neuro-Signals.

[35] Kerr J.R. (1999). Cell adhesion molecules in the pathogenesis of and host defence against microbial infection. Mol. Pathol..

[36] Tvaroska I., Selvaraj C., Koca J. (2020). Selectins-The Two Dr. Jekyll and Mr. Hyde Faces of Adhesion Molecules—A Review. Molecules.

[37] Lu H., Yang H.L., Zhou W.J., Lai Z.Z., Qiu X.M., Fu Q., Zhao J.Y., Wang J., Li D.J., Li M.Q. (2021). Rapamycin prevents spontaneous abortion by triggering decidual stromal cell autophagy-mediated NK cell residence. Autophagy.

[38] Yuan T., Deng M., Wang Y., Duan B., Chen Q., Fang Z., Li Q., Zhou G., Chen H., Wang Q. (2025). Combined cerebrospinal fluid sCD163, MMP-9, with serum NCAM1 protein levels for predicting the prognosis of patients with tuberculous meningitis. Sci. Rep..

[39] László Z.I., Bercsényi K., Mayer M., Lefkovics K., Szabó G., Katona I., Lele Z. (2020). N-cadherin (Cdh2) Maintains Migration and Postmitotic Survival of Cortical Interneuron Precursors in a Cell-Type-Specific Manner. Cereb. Cortex.

[40] Ito S., Kawauchi T. (2025). Roles of N-cadherin in cerebral cortical development: Cooperation with membrane trafficking and actin cytoskeletal regulation. Neural Regen. Res..

[41] Chen Y., Liu N., Yang Y., Yang L., Li Y., Qiao Z., Zhang Y., Li A., Xiang R., Wen L. (2024). NCAM1 modulates the proliferation and migration of pulmonary arterial smooth muscle cells in pulmonary hypertension. Respir. Res..

