# Supplementary material for: Synergistic Roles of InlA, InlB and LLO in the Infection of Trigeminal Ganglion Neurons by Ovine-Derived Listeria monocytogenes LM90SB2
Source: Animals (Basel). 2026 Apr 30;16(9):1383. doi: 10.3390/ani16091383 (PMC13162993; doi:10.3390/ani16091383)
Supplement: Supplementary file 1 [file animals-16-01383-s001.zip › animals-4180484-supplementary.pdf]

Table S1. Primer sequences used for the construction and verification of the InlABO mutant strain.

Primers	Squence（5'-3'）	Length/bp
llo-F1(Upstream arm)	**CGGGATCC**TGACATCGTTTGTGTTTGAGCCAG	512
llo-R1(Upstream arm)	TAATTTTTTATTACTTTTATTTCACTCTCCTTCTACAT	
llo-F2(Downstream arm)	ATGTAGAAGGAGAGTGAAATAAAAGTAATAAAAAATTA	458
llo-R2(Downstream arm)	**AACTGCAG**CAGCAGGGTTCCTTTTGGCTTGTA	
llo-A (Verification)	ACACTCGGACCATTGTAGTCATCT	3474
llo-B (Verification)	ATCAGCCCGCTCTACCTCGGAAAG	

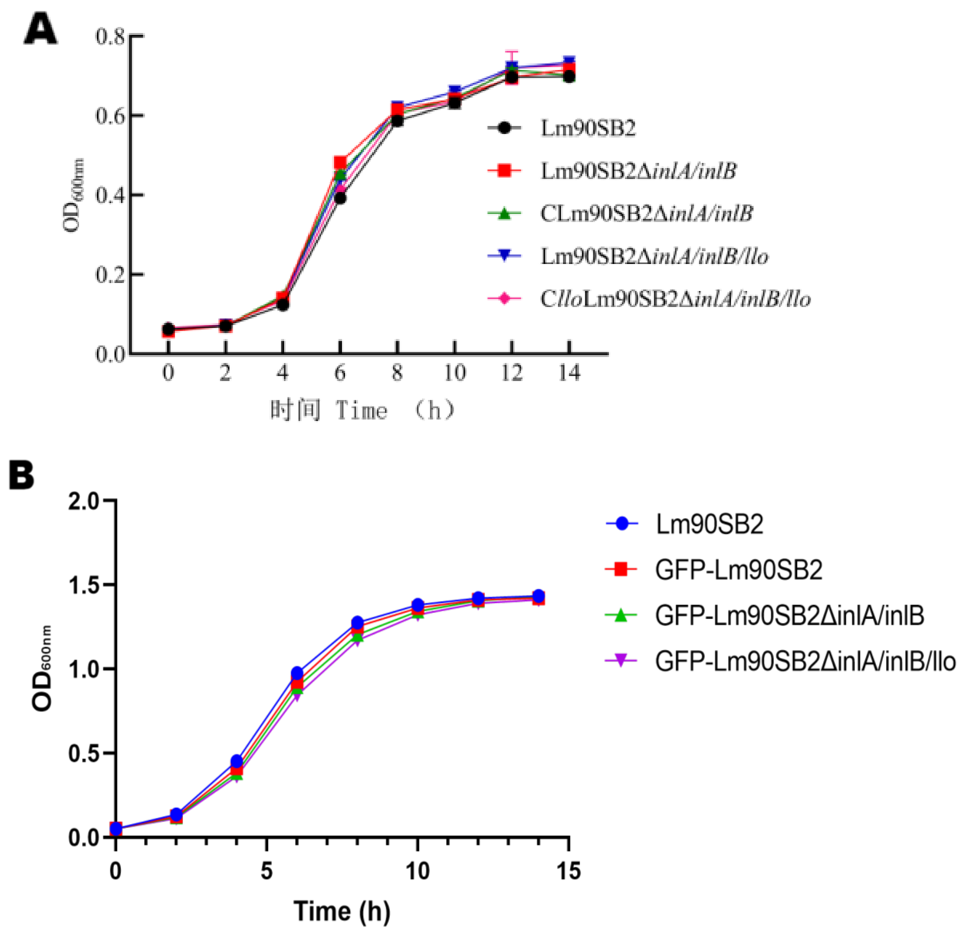

Supplementary Figure S1. Growth kinetics of the Listeria monocytogenes strains used in this study. (A) Growth curves of the wild-type LM90SB2, the double-gene deletion mutant ΔinlAB, and the triple-gene deletion mutant ΔinlABO. (B) Growth curves of the wild-type LM90SB2 and the corresponding sfGFP-labeled strains (GFP90, GFPAB, and GFPABO). All bacterial strains were cultured in BHI medium at 37°C. The optical density (OD600) was monitored over a 14-hour period. Data are presented as the mean ± SD of three independent experiments. As shown, the sequential

gene deletions and the introduction of the integrative sfGFP plasmid imposed only a negligible metabolic burden during the logarithmic phase. All strains eventually converged to identical peak densities in the stationary phase, confirming that these genetic manipulations did not significantly impair fundamental bacterial viability or carrying capacity.
